# Real‐Time Gas Chromatography System for Ultrasensitive Monitoring of Odorants in Natural Gas Infrastructure

**DOI:** 10.1155/ianc/8962523

**Published:** 2025-12-23

**Authors:** Zixun Chen, Kejing Song, Pu Zhang, Li Zhou, Zhenquan Tu

**Affiliations:** ^1^ Research Institute of Natural Gas Technology, PetroChina Southwest Oil & Gasfield Company, Chengdu, Sichuan, China, cnpc.com.cn; ^2^ Key Laboratory of Natural Gas Quality Control and Energy Measurement, State Administration for Market Regulation, Chengdu, Sichuan, China; ^3^ Key Laboratory of Natural Gas Quality Control and Energy Measurement, CNPC, Chengdu, Sichuan, China, cnpc.com.cn

**Keywords:** gas chromatography, natural gas (NG), odorants, photoionization detector

## Abstract

Natural gas (NG), primarily composed of methane, is colorless and odorless, thus necessitating odorization to enable leak detection and accident prevention. Precise monitoring of odorants is critical to meet safety standards while preventing excessive dosing. This study introduces a real‐time gas chromatography system integrating gas chromatography and photoionization detection for the real‐time, online odorant analysis in gas pipelines. This system effectively isolates tetrahydrothiophene (THT) and sulfur‐free odorants from NG, enabling accurate concentration quantification. It achieves THT detection within 2∼150 mg/m^3^, with a relative standard deviation (RSD) of less than 1.5% and maximum deviation of less than 3 mg/m^3^. Additionally, this system exhibits good field applicability. When combined with a cloud‐based warning platform, it can achieve monitoring, warning, and self‐optimization of odorant concentration. This study demonstrates the reliability of this system for NG odorant monitoring, providing a robust solution for pipeline safety management and regulatory compliance, while offering a new approach for NG safety testing.

## 1. Introductiona

Natural gas (NG) is a naturally occurring hydrocarbon gas mixture extracted from subsurface rock layers [1]. In recent years, NG, as a clean‐burning and primary source of energy, has been extensively used in multiple fields such as residential, commercial, industrial, and power generation, playing an important role in the development of industry and economy [2, 3]. NG mainly consists of hydrocarbons such as methane, along with minor amounts of carbon dioxide, water vapor, and traces of hydrogen sulfide [4–6]. As a result of its predominant component, NG is a colorless and odorless gas, making it challenging to detect in the event of a leak [7–9]. NG leaks have led to catastrophic accidents, such as the 1937 explosion at the New London School in Texas, which resulted from undetected leakage at a pipeline joint but went undetected [10]. Beyond the direct detection method of pipeline leaks [11, 12], odorization serves as a critical measure to identify leaks. By odorization, even minor leaks can be promptly detected, thereby ensuring timely intervention and mitigating potential safety risks. Therefore, the adequate odorization of NG is essential, prompting countries around the world to establish regulatory requirements for NG odorization to ensure timely detection of leaks.

The US Federal Regulations (49 CFR 192.625) mandate that distribution‐grade NG be odorized to a detectable concentration at one‐fifth of the lower explosive limit (LEL) in air, so that people can detect potential gas leaks with a normal sense of smell [13–15]. Canadian regulatory requirements stipulate that NG must be detectable at 20% of the LEL, equal to detectable odor at 1% NG in air by volume [10]. In China, GB 50028‐2020 *Design Specification for Urban Gas* and industry standard CJJ 148‐2010 *Technical Regulations for Odorization of Urban Gas* clearly stipulate that urban gas should have a noticeable odor, for example, the concentration of the odorant, such as tetrahydrothiophene (THT), must reach a minimum of 8 mg/m^3^ at the pipeline endpoint.

In order to rapid detect NG leaks, odorants added to NG must have a distinct indicative effect and some specific physicochemical properties, including strong unpleasant odor, chemical stability, high volatility, and nontoxicity during combustion [8, 16]. Currently, NG odorization employs two distinct odorant formulations to ensure reliable leak detection: sulfur‐containing compounds such as mercaptan compounds, THT, and dimethyl sulfide (DMS) [17], and sulfur‐free odorants such as methyl acrylate (MA) and ethyl acrylate (EA) [18, 19]. Among these, THT possesses the advantages of low cost, high efficiency, ease of use, and low pipeline corrosivity, commonly used in mainland Europe, China, and Japan [16].

The concentration of odorants requires strict regulation, as low concentrations compromise the odorization effect, while high concentrations pose the risk of air pollution and health hazards [20]. To supervise the concentration of the odorant within the target range after injection, it is necessary to select appropriate sensors to monitor the odorant concentration. On the one hand, since NG may contain a trace of sulfur‐containing compounds [21, 22], detection of sulfur‐based odorants in NG requires accurate differentiation between sulfur‐containing compounds and odorants. On the other hand, the key to odorant detection is the miniaturization, portability, and accuracy of the equipment. Currently, several different methods have been used to detect NG odorants, including olfactometry [15], detection tube method [23], ultraviolet (UV) spectroscopy [8], electrochemical sensor method [24], ion migration spectroscopy (IMS) [25], and gas chromatography (GC) [26] (as summarized in Table S1). The olfactometry (ASTM D6273–08) is based on the human sense of smell, and the procedures require trained individuals to “sniff at the apparatus exhaust” to measure odorization [27, 28]. However, odor detection thresholds vary from person to person, and this method is limited by significant measurement errors and potential health risk [29]. The detection tube method operates on a principle resembling that of a colorimetric tubes, where measurements rely on color changes. It is suitable for on‐site use, but costly for tube replacement and imprecise for visual observation errors. The electrochemical detection method has the advantage of rapid and easy quantitative detection, but the presence of hydrogen, oxygen, and sulfides would influence the detection results significantly. Furthermore, environmental conditions such as temperature and humidity may also affect the results. IMS operates by accessing the duration of ions passing through an electric field to detect samples [30], offering a low detection limit and swift analytical speed [31], yet limited by the high cost of the requisite equipment. GC utilizes different chromatographic columns and detectors to separate and detect distinct odorants, resulting in good separation and detection effects with high sensitivity and accuracy. Among them, gas chromatography with sulfur chemiluminescence detector (GC‐SCD) can only perform offline detection and requires manual sampling, which has the problems of sulfur adsorption, complicated operation and high cost [27, 32]. Gas chromatography with thermal conductivity detector (GC‐TCD) enables equipment portability and on‐site detection capability. However, its test results can be interfered from components such as sulfur compounds in the context of continuous online application. In summary, while numerous methods exist for the detection of odorants, the development of portable, user‐friendly, and online gas detectors for the quantitative monitoring of odorant compound concentrations remains an area necessitating further.

Photoionization detection (PID) method employs UV light generated by inert gas vacuum discharge to ionize the target gas molecules and determines the concentration of the gas by measuring the current intensity generated by the ionized gas [33, 34]. It is capable of measuring organic compounds at parts per billion (ppb) level, offering notable advantages of low detection limit, high precision, and low investment costs [35, 36]. Currently, PID finds extensive applications in the field of environmental testing and industrial safety [37–39]. Combined with chromatographic columns, PID can be very suitable for precise testing of odorants in NG. Moreover, the GC‐PID method has a complete calibration and traceability system, which facilitates the recognition of method detection results and is easy to promote. This approach represents a promising avenue for the online detection of odorants.

In this study, we developed a portable real‐time GC system that integrates a GC separation module and a photoionization detector for detecting NG odorants for the first time: THT, MA, and EA. By directly connecting this system to NG pipelines and combining it with a cloud‐based warning platform, real‐time monitoring of the concentration of NG odorants can be achieved, enabling early warning and self‐optimization. The main objective of this research is to develop an NG odorant monitoring system suited for mobile deployment, with a precision of up to 1.5% and deviation less than 2 mg/m^3^, thereby showcasing significant potential for application in NG leak detection.

## 2. Materials and Methods

### 2.1. The Real‐Time GC System

**Figure 1:**
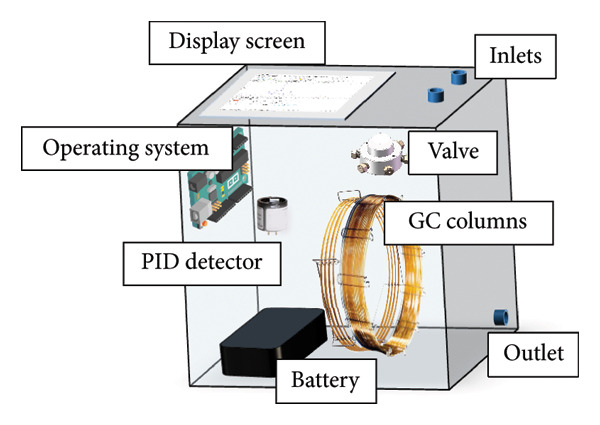
Schematic of the real‐time gas chromatography system.

The real‐time GC system is an integration of a customized software and hardware system, consisting mainly of chromatography columns, a programmed module, a data processing unit, a PID, valves, and a battery (Figure 1). As to the selection of chromatographic columns, a preseparation column (5 m in length, Agilent Technologies, USA) and a main separation (DB‐1701, 14% cyanopropyl phenyl and 86% dimethyl polysiloxane, 0.25 μm film thickness, 30 m in length × 0.32 mm i.d., Agilent Technologies, USA) are selected to separate odorants from other components (benzene series and heavy hydrocarbon components). The PID uses a UV lamp with a 10.6 eV ionization energy to avoid interference from numerous other substances. The valve rotation is programmed to ensure precise dosing of gas into the system, and the flow rate is accurately controlled by the electronic power control (EPC). The entire hardware system adeptly facilitates the extraction of the NG from the gas pipeline, as well as the separation and quantitative detection of odorant compounds.

### 2.2. System Operation

The real‐time GC system is versatile, accommodating a broad spectrum of applications via a sampling cylinder or directly from a gas pipeline. Upon initiation of the software and connection of the gas sample to the device’s inlet, the valve control allows for automatic sample collection. The sampling duration is also set through the software. Once the sampling phase is complete, the sample is propelled by the carrier gas into the chromatography column. As compounds elute from the GC column and enter the detector, methane, the main component of NG, does not respond in PID, while odorant is analyzed. When the analysis phase is complete, the raw data is processed by customized software, which automatically reports the quantitative concentration value for the odorant.

### 2.3. Analytical Method

A reference gas containing 7.70 mg/m^3^ THT (methane as supplementary gas) was used as an example of an optimized target concentration. The analysis process is as follows: after sampling, the sample gas enters the quantitative loop, column 1 and column 2 successively, with the carrier gas, through the valve rotation for separation, and ultimately entering the detector for analysis. Throughout the process, column 1 performs preliminary separation of the sample gas. Subsequently, heavy hydrocarbons are exhausted by switching the valve and reversing the flow of the carrier gas. The sample gas then proceeds into column 2 for deep separation of the odorant from benzene series and other impurities in NG. This integration of chromatography columns and analytical methodology expedites the analysis process. Prior to the development of this custom analytical method for the odorant detector, specific analytical parameters were configured in the software to achieve the target THT response with minimum sampling times. Selected optimized parameters are detailed in the Supporting Information (Table S2).

#### 2.3.1. Materials and Samples Preparation

Seven THT reference gases (methane as supplementary gas, from China Institute of Testing Technology) with approximate concentrations of 2, 5, 8, 20, 40, 100, and 150 mg/m^3^ were prepared for subsequent fitting of the instrument calibration curve. Gas mixtures containing sulfur compounds and hydrocarbons (from China Institute of Testing Technology) were prepared for analytical interference resistance in instrumentation. Detailed compositional specifications for these reference gases are provided in Tables 1 and 2.

**Table 1:** THT reference gases.

Cylinder number	1#	2#	3#	4#	5#	6#	7#
Content of THT (mg/m^3^)	1.98	4.77	7.70	19.6	38.9	99.8	148

**Table 2:** Multi‐component reference gases.

	Compound	8#	9#	10#
Content (mg/m^3^)	THT	8.892	—	—
	hydrogen sulfide	1.019	—	15.945
	carbonyl sulfide	1.078	—	28.107
	methyl mercaptan	1.112	—	22.358
	ethyl mercaptan	1.076	—	29.071
	carbon disulfide	—	—	36.337
	dimethyl sulfide	—	—	29.458
	dimethyl disulfide	—	—	44.661
	diethyl sulfide	—	—	35.633
	isopropyl mercaptan	—	—	35.634
	n‐butyl mercaptan	—	—	42.196
	tert‐butyl mercaptan	—	—	42.196
	methylthioethane	—	—	43.040
	thiophene	—	—	39.367

Content (mol/mol, ppm)	He	2002.191	—	—
	H_2_	1994.292	—	—
	O_2_	2006.055	—	—
	N_2_	30381.029	—	—
	CO_2_	30278.794	—	—
	C_2_H_6_	30023.173	—	—
	C_3_H_8_	10035.753	—	—
	i‐C_4_H_10_	991.162	—	—
	n‐C_4_H_10_	995.210	—	—
	neo‐C_5_H_12_	999.092	—	—
	i‐C_5_H_12_	995.019	—	—
	n‐C_5_H_12_	981.025	—	—
	C_6+_	998.730	—	—
	benzene	—	50.8	—
	toluene	—	50.7	—
	ethylbenzene	—	50.6	—
	o‐xylene	—	51.2	—
	m‐xylene	—	50.7	—
	p‐xylene	—	50.9	—

#### 2.3.2. Data Analytics

The response signals were studied to characterize the performance of the real‐time GC system. The data produced by PID appears as a continuous signal. After generating a spectrogram through the software, the *y*‐axis represents the signal intensity (measured in millivolts), while the *x*‐axis illustrates the retention time (RT, measured in minutes). Quantification of odorant concentration is accomplished by calculating the peak area corresponding to the specific RT of the odorant compound. To obtain a calibration model for predicting the concentrations of the odorant compounds, linear regression was employed on the data obtained from reference gases measurements [40, 41]. The sensitivity of this system can be inferred from the slope of the calibration curve, while the response values can be calculated using the formula Eg/Ea (Ea represents the signal intensity detected in ambient air and Eg denotes the signal intensity when the target gas is present). Once the parameters of the calibration curve (slope and *y*‐axis intercept) have been established, the peak areas of samples with unknown concentrations measured by this system can be input to obtain the concentrations of individual odorant compounds.

## 3. Results and Discussion

### 3.1. Analytical Capability

#### 3.1.1. Separation Performance

Reference gases of different concentrations and compositions were connected to the real‐time GC system, and the responses of this system to each gaseous substance are depicted in Figure 2. As shown in Figure 2(a), the retention time of THT in the chromatogram was 4.8 min, the peak shapes were basically symmetrical and narrow, and the half‐peak width was about 0.07 min (7.70 mg/m^3^ THT). The entire analysis was completed within 8 min. The detector exhibited a concentration‐dependent response profile, with signal intensity increasing proportionally to THT concentration in introduced reference gases. At a THT concentration of 7.70 mg/m^3^, the detector generated a signal intensity of 169 mV, accompanied by a response ratio (Eg/Ea) of 1.3 [10]. The system demonstrates a selective responsiveness to THT.

Figure 2(a) Response of real‐time gas chromatography system to different concentrations of THT. (b) The response to multicomponent gas mixtures.

**Figure:**
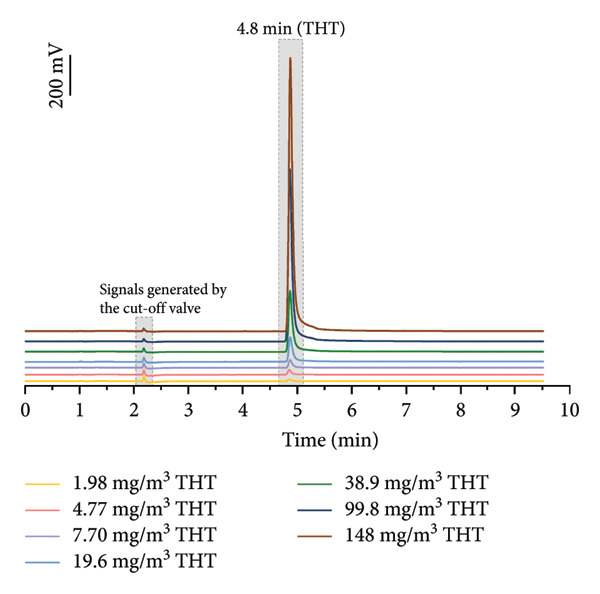
(a)

**Figure:**
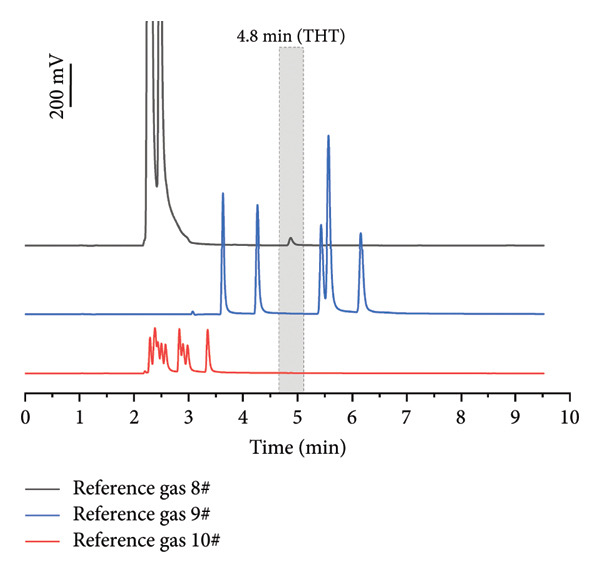
(b)

To simulate actual application in pipelines, the methane‐based gas mixture containing sulfur compounds and hydrocarbons was injected into the real‐time GC system (Figure 2(b)). Because the UV lamp energy of the PID in this portable detection device is 10.6 eV, which is higher than the ionization potential energy of the main sulfur‐containing compounds in NG (hydrogen sulfide: 10.46 eV, methyl mercaptan: 9.45 eV, ethyl mercaptan: 9.31 eV), and some C4+ heavy hydrocarbons (C4+) in NG (Table S3) [42]. These molecules can be ionized by the UV lamp in PID, generating electrical signals and forming spectral peaks [33]. Other compounds cannot be ionized by the UV lamp and do not produce a signal to affect the spectrum.

In addition, suitable GC columns facilitated the baseline separation of THT, and the signals generated by interfering compounds and the cut‐off valve appeared within 2–3 min, confirming the negligible analytical interference. Although more sulfur compounds and aromatics in NG produced signals in the detector (Figure 2(b)), these peaks were completely separated from the peak of THT, indicating that these substances would not interfere with the detection of THT [43]. Consequently, GC‐PID exhibits exceptional selectivity for THT, verifying the robustness of the system for odorant detection in complex mixtures.

#### 3.1.2. Analytical Accuracy

In order to verify the analytical accuracy of this system, a calibration curve (peak area vs. the concentration) was plotted based on the test results of seven THT reference gases (Figure 2(a)) and is shown in Figure 3.

Figure 3Calibration curves (peak area vs. concentration) of detectors in the system: (a) 2 to 20 mg/m^3^; (b) 20 to 150 mg/m^3^.

**Figure:**
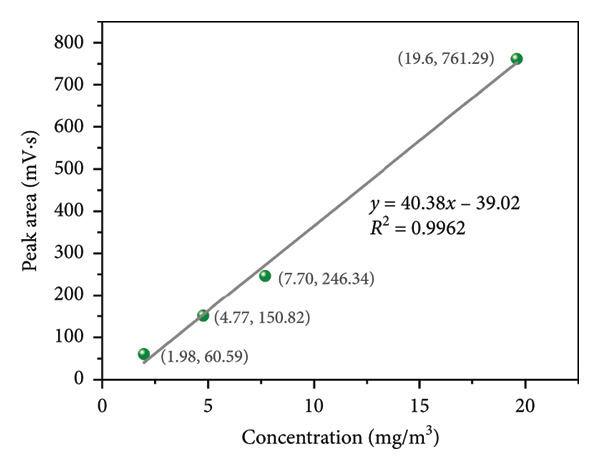
(a)

**Figure:**
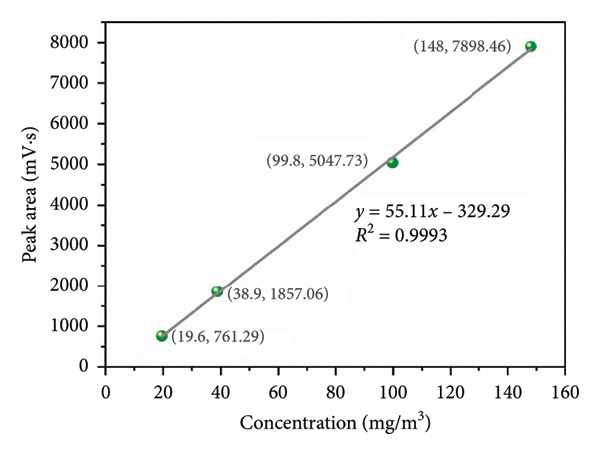
(b)

Sulfur compounds are always calibrated using a single calibration curve [44]. However, given that the concentration range of THT spanned over 50‐fold, a segmented calibration strategy was adopted. This ensured the accuracy of actual calibration results and the practicality of the calibration curve. So, the calibration curves were drawn in segments. Therefore, the experimental results were divided into two parts: low concentration (2–20 mg/m^3^, Figure 3(a)) and high concentration (20–150 mg/m^3^, Figure 3(b)). Four concentration points were used for calibration to avoid the analytical error caused by single‐point calibration. The calibration curves were linear, and the THT concentration showed a good linear relationship with the peak area (*R* ^2^ > 0.99). At this point, the sensitivities of the detector, calculated from the slopes of the calibration curves, were 40.38 and 55.11, respectively. The calibration curves formed by the segmented linear relationship of multiple concentration points were used as a benchmark for predicting the unknown concentrations of THT, which ensured the accuracy of the PID detector for the measurement of THT content within the range of 2∼150 mg/m^3^.

**Figure 4:**
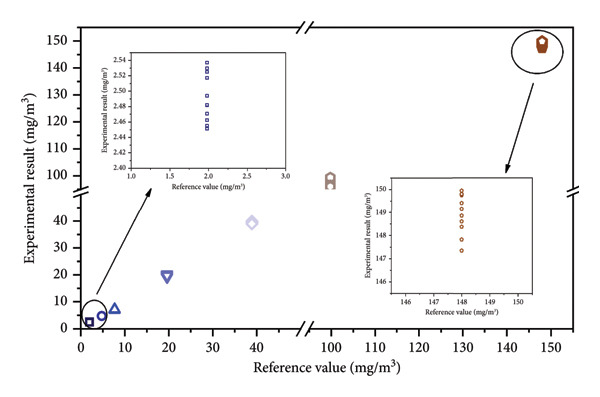
Comparison of experimental results and the reference values of THT at different concentrations.

Combined with the segmented calibration curve, THT at different concentrations has been detected several times. The THT content values output by the software were collected and compared with the reference values, and the results are displayed in Figure 4 and Table 3.

**Table 3:** Comparison of experimental results and the reference values of THT at different concentrations.

Reference value (mg/m^3^)	Calibration curve	Experimental results (mg/m^3^)											Average result (mg/m^3^)	RSD (%)	Absolute deviation (mg/m^3^)	Relative deviation (%)
		1	2	3	4	5	6	7	8	9	10	11				
1.98	*y* = 40.38*x* − 39.02	2.54	2.53	2.52	2.46	2.52	2.45	2.49	2.47	2.48	2.48	2.46	2.49	1.25	0.51	25.7
4.77		4.71	4.74	4.75	4.73	4.71	4.71	4.72	4.74	4.71	4.72	4.70	4.72	0.32	0.05	1.0
7.70		7.11	7.09	7.05	7.00	7.05	7.06	7.07	7.08	7.09	7.09	7.18	7.08	0.64	0.62	8.1
19.6		19.71	20.25	20.05	19.97	19.85	20.00	19.76	19.15	19.71	19.61	19.72	19.80	1.44	0.20	1.0

19.6	*y* = 50.11*x* − 329.29	19.74	20.14	19.99	19.93	19.84	19.95	19.78	19.33	19.74	19.67	19.74	19.80	1.06	0.20	1.0
38.9		39.66	39.81	39.86	39.74	39.71	39.62	39.60	39.65	39.63	39.74	38.98	39.64	0.58	0.74	1.9
99.8		96.32	96.78	97.20	97.68	98.03	98.56	99.00	99.36	96.57	95.77	96.00	97.39	1.26	2.41	2.4
148		148.87	147.35	147.83	148.38	148.62	149.15	149.40	149.74	149.94	149.77	149.80	148.99	0.58	0.99	0.7

The average values of multiple detections of seven bottles of THT reference gas were 2.49, 4.72, 7.08, 19.80, 39.64, 97.39, and 148.99 mg/m^3^. According to the formula of relative standard deviation (RSD) = $\sqrt{\left(\sum {\left(x_{i}-x\;\right)}^{2}\right)/\left(n-1\right)}/x\times 100\%$ [33], RSDs of the detection results using this system were all less than 1.5%. At low THT content (2–20 mg/m^3^), the experimental results based on the calibration curve were very similar to the reference value, with absolute deviations below 1 mg/m^3^. At a higher THT content (20–150 mg/m^3^), the absolute deviations were slightly increased, but the relative deviation was always lower than 3%.

In practice, to ensure the accuracy of the system, it is necessary to select the graph points of the calibration curve according to the actual concentration range to be detected, so that the accuracy and consistency of the system can reach a higher level.

The system with established calibration curves was used to detect a gas mixture containing 19.8 mg/m^3^ THT, and the results are shown in Table 4. After seven tests, the RSD of this system is less than 2%. Compared with the test results of traditional devices using GC‐TCD and GC‐SCD, this system exhibits better repeatability, which increased by over 40%.

**Table 4:** The experimental results of THT using different method.

Detection method	Experimental results (mg/m^3^)							RSD (%)
GC‐TCD	20.34	19.53	20.07	21.16	20.89	20.07	19.80	2.87
GC‐SCD	20.80	20.49	19.56	18.66	19.33	19.46	19.18	3.82
This system	19.37	19.34	19.15	19.89	19.68	18.97	19.19	1.64

### 3.2. Application in Sulfur‐Free Odorants

**Figure 5:**
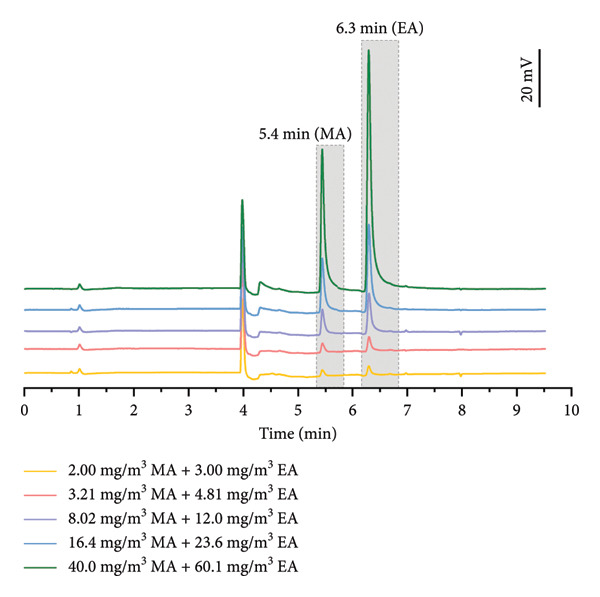
Response of the system to different concentrations of MA and EA.

The real‐time GC system can be further adapted for the detection of sulfur‐free odorants (MA and EA). Figure 5 shows the chromatographic separation and analytical efficacy of the system. As shown in the chromatogram in Figure 5, the separation degree of MA and EA exceeded 7, which means they were efficiently separated by the column and can be individually quantified via distinct chromatographic peaks. By changing the analytical method, this system can quantitatively detect different types of odorants. Through precise modulation of valve switching times, the separation process has been adjusted, thereby expanding the versatility of the system to achieve quantitative detection of diverse odorants.

### 3.3. Field Implementation

The real‐time GC system has been implemented across 7 urban distribution terminals (including two industrial users and five residential users) in south‐western China. Sampling ports were located on the gas pipelines and directly connected to the inlet of the system. Before the system connected to the pipeline, the calibration curves had already been plotted for the purpose of subsequent data output. After each analytical cycle completed, data were automatically reported and transmitted via software. Weekly calibrations ensure measurement reliability post‐installation. When the relative deviation between weekly calibration results and the first calibration exceeds 10%, the calibration curve shall be redrawn. When the calibrated peak area falls below 400 mV·s, it indicates that the UV lamp has severely degraded and requires replacement.

A representative industrial application case is presented below (Figure 6(a)). The pipeline operated at 12,500 kPa and 15°C, with the sampling site located 12 km downstream from the odorant injection point. THT was pulse‐injected at a nominal 20 mg/m^3^. Over 41 days of continuous monitoring, measured THT concentration exhibited oscillation between 11.01 mg/m^3^ and 33.75 mg/m^3^ (Figure 6(b)), attributable to dynamic variations in gas flow velocity, temperature, and pressure along the pipeline. During this field implementation process, the real‐time GC system was calibrated weekly using a gas mixture containing 19.8 mg/m^3^ THT. Each calibration involved three consecutive tests, and the test results are shown in Table 5. The system stability (*S*_*t*_) can be calculated according to the formula $S_{t}=\left(\left(\rho_{2}-\rho_{1}\right)/\overset{¯}{\rho }\right)\times 100\%$, where *ρ* is the result of each calibration. The calculation results indicate that the system’s calibration outcomes for the same gas mixture remained consistent weekly. The stability data for consecutive weeks were 0.77%, 2.68%, and 2.86%, consistently below 3.00%. In addition, no significant changes were observed in the peak area of these four calibration tests (Table S4), indicating that the UV lamp did not degrade noticeably during this period. It is evident that the system demonstrated sustained operational stability throughout the month‐long field implementation, accurately reflecting the changes of THT in pipelines. The performance of the system has also been further validated through the field implementation.

Figure 6(a) An example of the spectrum actually detected by this system. (b) The test results detected by this system for 1 month at a test point. (c) The test results after adjusting the odorant dosage. (d) Warning process of the real‐time GC system and schematic diagram of optimization system for odorants.

**Figure:**
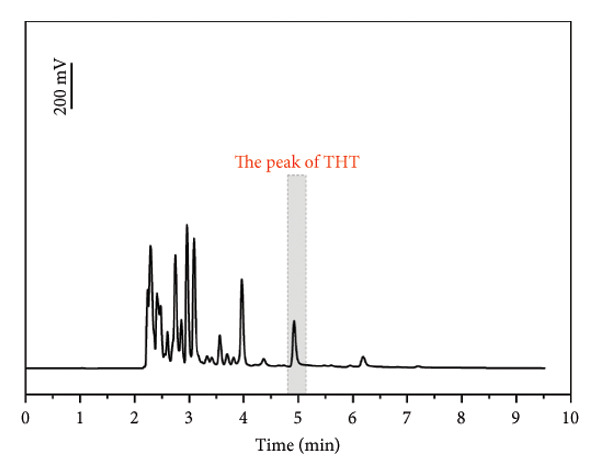
(a)

**Figure:**
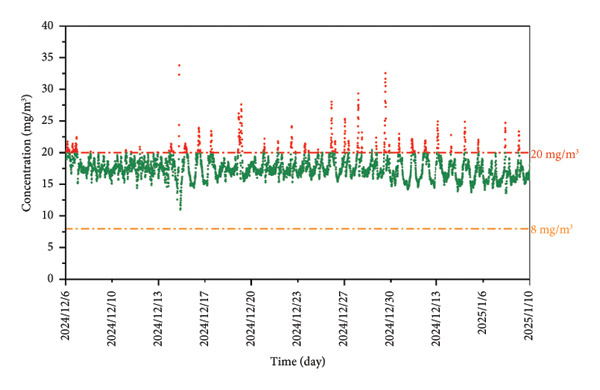
(b)

**Figure:**
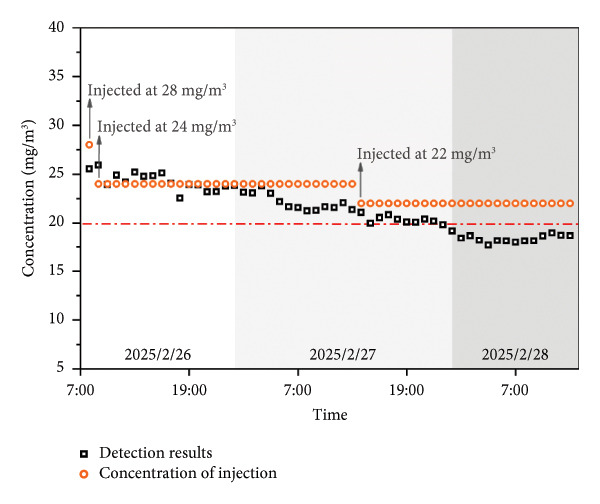
(c)

**Figure:**
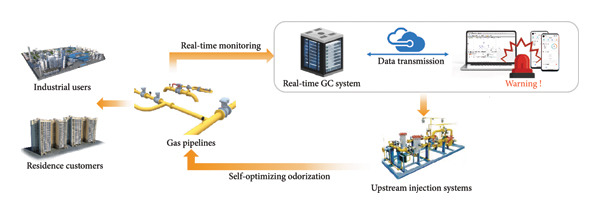
(d)

**Table 5:** The calibration results of the real‐time GC system for 19.8 mg/m^3^ THT in field implementation.

	Calibration date	Experimental results (mg/m^3^)			Average result (mg/m^3^)
1st Calibration	Dec. 13	20.20	20.77	20.83	20.60
2nd Calibration	Dec. 20	21.00	20.64	20.63	20.76
3rd Calibration	Dec. 27	20.38	20.20	20.06	20.21
4th Calibration	Jan. 3	19.85	19.47	19.60	19.64

To ensure the odorant content in compliance with the local legislation threshold (8∼20 mg/m^3^) [45], a cloud‐based warning platform was integrated into the real‐time GC system. The final test results were uploaded to the cloud‐based platform in real time. When concentrations deviated from target ranges, this system triggered alerts and sent a message to the mobile phone. Administrators can also monitor the data in real time, adjust the amount of odorant dosing according to the data, and view the data feedback in real time after adjustment. Figure 6(c) shows the changes in test data after adjusting the odorant dosage. The test results were reported every hour. As the concentration of injection decreased, the test results subsequently decreased until they gradually smoothed out. Due to the distance between the sampling site and the odorant injection point, a delay of approximately 1 to 2 hours was observed in the correction process. Furthermore, it took more than 10 hours for the concentration to stabilize. When upstream injection systems were also dynamically connected to the cloud‐based platform, the real‐time GC system can achieve real‐time monitoring and self‐optimization of THT content, thereby maintaining regulatory compliance.

### 3.4. Comparison

Equipment manufacturers have used various methods to detect odorants. A comprehensive comparison between this real‐time GC system and devices using other methods is presented in Table 6. Compared to other methods, this real‐time GC system has the advantages of a low detection limit and low cost, although its analysis speed is marginally inferior. This system is adapted to various application scenarios, indicating its considerable potential.

**Table 6:** Comprehensive comparison of this real‐time GC system and other methods.

Method	Detection limit (mg/m^3^)	Analysis time	Estimated cost	Application
GC‐PID (this real‐time GC system)	0.4	about 8 min	About $35000	online detection, offline detection, portable device
GC‐IMS [25, 30]	0.4	about 8 min	About $210000	offline detection, portable device
GC‐TCD	4	about 4 min	About $85000	offline detection, portable device
GC‐μTCD [46, 47]	2	about 2 min	About $105000	offline detection, portable device
GC‐SCD [45, 48]	0.5	about 17 min	About $100000	offline detection

*Note:* Related information is derived from equipment manufacturers and relevant literature.

## 4. Conclusion

A real‐time GC system was developed for in situ quantification of odorants in NG infrastructure. By synergistically integrating a GC system and a photoionization detector, this system achieves a breakthrough in overcoming limitations of traditional detection methods regarding real‐time performance, portability, and automation. This system provides a real‐time automatic detection of NG odorant compounds within a 10‐min analytical cycle. In laboratory validation, the system demonstrated exceptional linearity (*R* ^2^ ≥ 0.99) within the THT concentration range of 2∼150 mg/m^3^, with method repeatability surpassing conventional benchtop systems by over 40%. As field implementation, this system demonstrated excellent operational stability and online capability. Combined with a cloud‐based warning platform, this system provides a viable pathway for self‐optimizing odorant dosing.

Overall, the success of this work demonstrates that a promising detection system has been successfully established, which has achieved significant progress in portability, rapid detection, repeatability, and integration. Its application potential can be further expanded through future research. For instance, replacing the chromatographic column with a suitable micro GC column could advance hardware miniaturization and enhance deployment flexibility. Expanding the range of detectable compounds would enable monitoring of a wider variety of odorants. Exploring deep integration with machine learning and building an intelligent monitoring platform can further improve the operational reliability and intelligence of NG infrastructure.

## Disclosure

All authors have read and agreed to the published version of the manuscript.

## Conflicts of Interest

The authors declare no conflicts of interest.

## Author Contributions

Zixun Chen: conceptualization, investigation, data acquisition, data analysis, writing–original draft and review and editing, and final approval. Kejing Song: investigation, data acquisition, writing–review and editing, and final approval. Pu Zhang: supervision, project administration, funding acquisition, data analysis, writing–review and editing, and final approval. Li Zhou: supervision, project administration, funding acquisition, and final approval. Zhenquan Tu: writing–review and editing and final approval.

## Funding

This work was supported by PetroChina Southwest Oil & Gas fied Company, with the project title “Research on the Mechanism and Design Preparation of Chromatographic Stationary Phase Separation for Natural Gas Sulfide Analysis (2024D108‐01‐01)” and “Research and Application of Key Technologies and Equipment for Detecting the Concentration of Gas Odorants (2024D105‐01–12).”

## Supporting Information

Supporting Information 1. Table S1: Comparison of different methods to detect NG odorants.

Supporting Information 2. Table S2: Analysis parameters of the device operation.

Supporting Information 3. Table S3: Ionization potentials (IPs) of common compounds in natural gas.

Supporting Information 4. Table S4: Calibration results of the real‐time GC system for 19.8 mg/m^3^ THT in field implementation.

## Supporting information


**Supporting Information** Additional supporting information can be found online in the Supporting Information section.

## Data Availability

The data used to support the findings of this study are available from the authors upon request.
