# Supplementary material for: Real‐Time Gas Chromatography System for Ultrasensitive Monitoring of Odorants in Natural Gas Infrastructure
Source: Int J Anal Chem. 2025 Dec 23;2025:8962523. doi: 10.1155/ianc/8962523 (PMC12752817; doi:10.1155/ianc/8962523)
Supplement: Supplementary file 1 — Supporting Information Additional supporting information can be found online in the Supporting Information section. [file IANC-2025-8962523-s001.docx]

Supporting information

Real-Time Gas Chromatography System for Ultra-Sensitive Monitoring of Odorants in Natural Gas Infrastructure

**Zixun Chen^1, 2, 3^, Kejing Song^1, 2, 3^, Pu Zhang^1, 2, 3^, Li Zhou^1, 2, 3^, Zhenquan Tu^1, 2, 3^**

*^1^Research Institute of Natural Gas Technology, PetroChina Southwest Oil & Gasfield Company, Chengdu, Sichuan, 610213, PR China*

*^2^Key Laboratory of Natural Gas Quality Control and Energy Measurement，State Administration for Market Regulation, Chengdu, Sichuan, 610213, PR China*

*^3^Key Laboratory of Natural Gas Quality Control and Energy Measurement, CNPC, Chengdu, Sichuan, 610213, PR China*

Table S1: Comparison of different methods to detect NG odorants.

Table S2: Analysis parameters of the device operation.

Table S3: The ionization potentials (IPs) of common compounds in natural gas.

Table S4: The calibration results of the real-time GC system for 19.8 mg/m^3^ THT in field implementation.

Table S1: Comparison of different methods to detect NG odorants.

| The method | Principle | Character | Standards |
| --- | --- | --- | --- |
| olfactometry | Odorant detection by nose | easy to operate,but with significant errors and health hazards | ISO/CD 18222 |
| detection tube method | Odorant detection based on color change of the medium after dissolution of the sample gas (iodometric or methyl blue method) | Small size, easy to operate, fast measurement speed, suitable for front-line staffs. High cost and large visual observation error. measurement results can only be used as a reference. |  |
| Ultraviolet spectroscopy | Odorant reacts with the chromogenic reagent to produce a specific substance, which undergoes electron transitions when exposed to ultraviolet light, producing an absorption spectrum | Optical technique, usability, robustness and wide analysis concentration range(from 10^–3^ to thousands  mg/m^3^). |  |
| electrochemical sensor method | Odorant undergoes redox reactions between the electrode and the electrolyte, outputs a current signal in the external circuit and displays concentration information | Fast and portable, suitable for field, large errors, different saples require different electrochemical sensors. | ASTM D7493-2008 |
| ion migration spectroscopy (IMS) | Odorouts undergo ionization to produce ions, which are separated and qualitatively determined based on the difference in migration time of the ions in the electric field | Low detection limit, fast detection speed, no need to separate samples, high equipment cost. Mainly used for detecting sulphur-free odorouts. |  |
| gas chromatography (GC) | Different components have different partition coefficients/polarities/boiling points and have different retention times in the columns | High precision, good separation; need to be quantified by external standard. Usually used for off-line detection | ISO 19739: 2004；ASTM D7493-2008 |

Table S2: Analysis parameters of the device operation.

|  | Analysis parameter |
| --- | --- |
| Carrier gas | N_2_, volume fraction ≥ 99.999% |
| Quantitative loop | 0.5 mL |
| Column temperature | 65 ℃ |
| Detector temperature | 45 ℃ |
| Column pressure | 14.5 psi |
| Column flow rate | 3.5 ml/min |
| Column 1 | DB-1701, 5 m×0.32 mm×0.5 μm |
| Column 2 | DB-1701, 30 m×0.32 mm×0.5 μm |

Table S3: The ionization potentials (IPs) of common compounds in natural gas.

| Compound | ionization potentials (eV) |
| --- | --- |
| Tetrahydrothiophene (THT) | 8.38 |
| ethyl acrylat (C_5_H_8_O_2_) | 10.3 |
| methyl acrylate (C_4_H_6_O_2_) | 9.9 |
| hydrogen sulfide (H2S) | 10.46 |
| carbonyl sulfide (COS) | 11.18 |
| Methyl mercaptan (CH_3_SH) | 9.45 |
| ethyl mercaptan (C2H5SH) | 9.31 |
| carbon disulfide (C_2_S) | 10.07 |
| He | 24.59 |
| H_2_ | 13.60 |
| O_2_ | 13.62 |
| N_2_ | 14.53 |
| CO_2_ | 13.77 |
| CH_4_ | 12.61 |
| n-C_4_H_10_ | 10.53 |
| C_5_H_10_ | 10.33 |
| neo-C_5_H_12_ | 10.30 |
| i-C_5_H_12_ | 10.32 |
| n-C_5_H_12_ | 10.28 |
| n-C_6_H_14_ | 10.13 |
| C8H16 | ≤9.98 |
| C₆H₁₂ | 9.88 |
| C_8_H_18_ | 9.80 |
| C_6_H_12_ | 9.70 |
| C6H6 | 9.24 |
| C7H8 | 8.83 |
| C_8_H_10_ | ≤8.77 |

Table S4: The calibration results of the real-time GC system for 19.8 mg/m^3^ THT in field implementation.

|  | calibration date | Peak area (mV·s) | | | Average peak area (mV·s) |
| --- | --- | --- | --- | --- | --- |
| 1st Calibration | Dec. 13 | 783.93 | 815.34 | 818.65 | 805.98 |
| 2nd Calibration | Dec. 20 | 828.02 | 808.18 | 807.63 | 814.79 |
| 3rd Calibration | Dec. 27 | 793.85 | 783.93 | 776.22 | 784.48 |
| 4th Calibration | Jan. 3 | 764.64 | 743.70 | 750.87 | 753.07 |
